# Application of the LEG NUI score to assess revision success in established distal femur non-unions

**DOI:** 10.1016/j.jor.2025.07.001

**Published:** 2025-07-07

**Authors:** Benedikt J. Braun, Carla Rau, Tanja Maisenbacher, Steven C. Herath, Mika FR. Rollmann, Maximilian M. Menger, Tina Histing, Marie Reumann

**Affiliations:** University Hospital Tuebingen, Eberhard-Karls-University Tuebingen, BG Unfallklinik, 72076, Tuebingen, Germany

**Keywords:** Fracture healing, Delayed healing, Leeds-Genoa Non-Union Index

## Abstract

**Background:**

The incidence of non-union following distal femur fractures is high. Management of persis-tent non-unions is challenging, often requiring multiple revision surgeries, thereby increasing patient morbidity and socioeconomic burden. Identifying patients at high risk for persistent non-union after revision is therefore crucial. The LEG NUI Score was originally developed to predict the need for early intervention following primary fixation of distal femur fractures. This study aimed to evaluate the LEG NUI Score's ability to predict the need for further revision surgery in a cohort of patients with established distal femur non-union undergoing their index revision procedure.

**Patients and methods:**

45 patients with complete clinical data were identified from a non-union database. The LEG NUI Score was calculated for the index revision procedure and assessed in relation to the healing out-come of the revision treatment. Comparative statistics and test characteristics were assessed, as well as a receiver operator characteristic analysis was performed. Only patients with uneventful healing after the first treatment were considered healed for the score calculation. Patients requiring more than one surgery, including second step masquelet were considered non-healers.

**Results:**

The union rate (healed non-unions) after the first non-union revision surgery was 55.5 %. 24.4 % of patients had an infected non-union. The average LEG NUI Score in patients with non-union healing was significantly lower than non-healing patients (3.36 ± 1.80 vs. 4.90 ± 1.21; p < 0.05). The AUC in the ROC Analysis was 0.755.

**Discussion:**

The LEG NUI Score shows potential applicability in the setting of revision surgery for established distal femur non-unions. Calculating the score may help surgeons identifying patients at in-creased risk for requiring subsequent surgical procedures, thus warranting closer postoperative surveillance. Further validation in larger cohorts is required to fully elucidate its clinical utility in this context.

**Level of evidence:**

Level III, Retrospective Cohort Study.

## 1 Introduction

The rate of non-union in distal femoral fractures is reported to be between 20 and 5 %, depending both on the biological and mechanical risk profile, as well as the status of infection.^1^, ^2^, ^3^ Associated with this is a high rate of both personal, as well socioeconomic burden of disease.^4^^,^^5^ Late detection, increased time-off-work and recalcitrant non-union requiring repeat hospitalization and higher length of stay further increase these negative effects.^6^^,^^7^ After the diagnosis of a distal femur non-union has been made treatment depends on the nature of the non-union, as well as the local status of infection.^8^, ^9^, ^10^, ^11^ The identification of patients at risk for repeat surgery is of critical importance in this cohort of patients with a high complication and persisting non-union rate.^11^^,^^12^

To address the diagnostic delay different scoring measures have been proposed to identify patients at risk for a delayed healing course early during the primary treatment of lower extremity fractures.^13^, ^14^, ^15^ Of these the Leeds-Genoa Non-Union Index (LEG NUI) Score has shown the highest sensitivity and specificity, as well as positive and negative predictive values.^13^^,^^14^ It is composed of 8 risk factors. These parameters include the post-surgical fracture gap, presence of infection, suboptimal mechanical stability of the fixation, initial fracture displacement (greater than 75 % of the shaft width), fracture site (only tibia being higher risk than femur scoring one point), significant soft tissue damage (open fractures or closed fractures with degloving), the use of an open reduction surgical technique, and the type of fracture (wedge or complex patterns). By evaluating these factors, the score aims to quantify the likelihood of non-union developing within the initial months after surgery. Each parameter is scored with one point if positive and a score ≥5 is associated with a higher non-union risk.

Based on the high predictive capabilities in the primary treatment setting our aim was to evaluate the scores’ ability to assess the need for further revision surgery in a cohort of patients with established distal femur non-union undergoing their index revision treatment.

## 2 Materials and methods

### 2.1 Patients

Patients treated for established distal femoral non-union were identified from a local non-union database (continuous enrollment, start date February 2009). Inclusion criteria were: diagnosis of established non-union of the distal femur, undergoing revision treatment at our institution, availability of complete clinical and radiographic data allowing for calculation of the LEG NUI Score and subsequent clinical and radiographic follow-up for a minimum of 6 months post-revision to determine the healing outcome. Patients lacking complete datasets for score calculation or adequate follow-up were excluded. The study was conducted in compliance with the Declaration of Helsinki and received approval from the responsible local Ethics Committee (Nr. 850/2019BO2). All patients consented to their surgical treatment. As only retrospective data was collected in accordance with the ethics committee decision, no separate consent for inclusion in the study database was required.

### 2.2 Methods

The LEG NUI Score was calculated for the index revision procedure. Only patients with uneventful healing after the first treatment were considered healed. Patients requiring more than one surgery, including second step Masquelet, were considered non-healers.

### 2.3 Methods of assessment

Healing outcome was determined based on clinical and radiographic follow-up for a minimum of 6 months post-revision.

### 2.4 Statistical analyses

Statistical analyses were performed using jamovi (version 2.6.26.0, The jamovi project). Descriptive statistics were calculated for patient demographics and the LEG NUI Score. Mean LEG NUI scores were compared between patients who achieved union and those with persistent non-union following revision treatment using the Mann-Whitney *U* test, based on data distribution assessment. Diagnostic test characteristics were determined for relevant score thresholds. Receiver operating characteristic (ROC) curve analysis was conducted to evaluate the discriminatory ability of the LEG NUI Score in predicting persistent non-union, and the area under the curve (AUC) was calculated. A p-value <0.05 was considered statistically significant.

## 3 Results

**Fig. 1:**
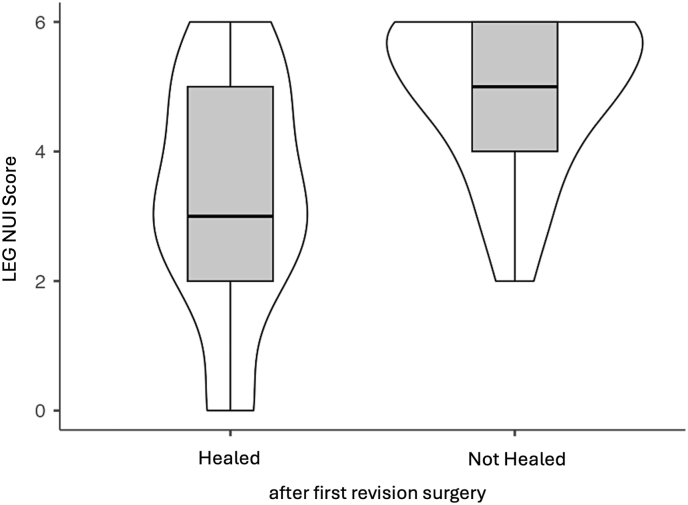
Boxplots visualizing median, interquartile range and min/max of the LEG NUI Score for patients that healed (left) and did not heal (right) after the first surgical revision. The violin elements depict the distribution of score counts for both groups.

A total of 45 patients were included in the final analysis. The mean patient age was 54.6 years (range: 21–88 years), with 19 females and 26 males represented in the cohort. The overall radiographic union rate following initial surgical revision treatment was 55.6 % (25/45). The primary analysis revealed a significant difference in preoperative LEG NUI Scores between outcome groups. Patients who achieved union demonstrated a significantly lower mean score compared to those with persistent non-union (3.36 ± 1.80 vs. 4.90 ± 1.21; p < 0.05) (Fig. 1).

**Fig. 2:**
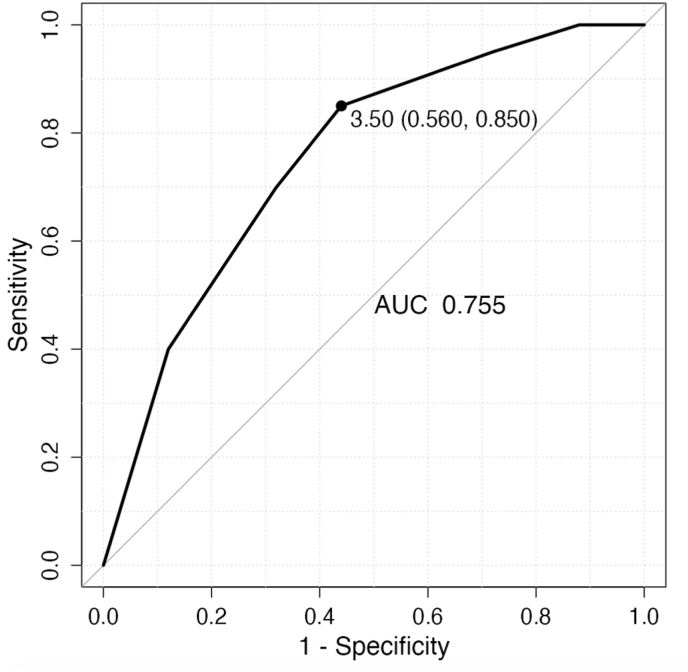
The ROC curve for the LEG NUI Score applied in the non-union revision setting. AUC, as well as test characteristics at the best cutoff (Youdens Index) are displayed (sensitivity = 0.850, specificity = 0.560). .

Receiver operating characteristic (ROC) analysis of the LEG NUI Score's ability to predict persistent non-union yielded an area under the curve (AUC) of 0.755 (Fig. 2). Applying the previously established risk threshold of a LEG NUI Score ≥5^13^^,^^14^ the sensitivity for predicting persistent non-union in this cohort was 0.700, and the specificity was 0.680 %. For this cutoff, the positive predictive value (PPV) was 0.636, and the negative predictive value (NPV) was 0.739. Further analysis using the Youden index identified an optimal LEG NUI Score cut off of 3.5 within this study population. At this threshold, the sensitivity for predicting persistent non-union was 0.850, with a corresponding specificity of 0.560.

## 4 Discussion

This study investigated the applicability of the Leeds-Genoa Non-Union Index (LEG NUI), a scoring system initially developed for predicting outcomes after primary fracture fixation in the tibia and femur,^13^ in the context of revision surgery for established distal femur non-unions. Our findings indicate that the preoperative LEG NUI Score was significantly lower in patients who achieved union after their index revision surgery compared to those who did not. The score demonstrated fair to good discriminatory ability in predicting the outcome of the index revision, with an Area Under the Curve of 0.755 in the Receiver Operating Characteristic analysis.

The positive finding of the association between the LEG NUI Score and healing outcomes after index revision surgery suggest that the risk factors that make up the score remain relevant also in a patient population with already established non-unions. The AUC indicates that the score performs markedly better than chance in distinguishing between patients likely to heal versus those likely to fail after the initial revision attempt. Using the established literature threshold of ≥5,^13^^,^^14^ the score yielded a sensitivity of 0.700 and a specificity of 0.680 in our cohort. This suggests a moderate ability to correctly identify patients who will go on to persistent non-union requiring further procedures (true positives) and those who will heal (true negatives) using this predefined cut off. These values are lower than in the original publication and also subsequent applications of the score. Initially the score was described with a high AUC in the ROC Analysis of 0.924, as well as a sensitivity of 91 % and specificity of 77 %. When the score is applied on their cohort by authors from an external institution outside of the initially describing study group in tibial shaft fracture cases, the scores Sensitivity (58.54 %), Specificity (91.07 %), PPV (75.31 %) and NPV (83.27 %) were described lower, highlighting the overall performance of the score, but also the potentially limited application in external settings.^16^ In our setting of an external application of the score on a subset of distal femoral fractures this limitation holds true, presenting with different fracture and non-union rates, than femoral and tibial shaft fractures, where the score was initially described.^13^^,^^17^^,^^18^ Furthermore, some of the criteria of the score have a higher base likelihood of being true, due to the nature of the most common revision surgeries.^11^^,^^19^ Most of the revision procedures entail some form of bone grafting requiring open surgery and are associated with bone defects, or fracture gaps above 4 mm, thus scoring higher in the LEG NUI.

Thus, while the LEG NUI score demonstrates potential utility, its moderate predictive performance (AUC 0.755, balanced sensitivity/specificity around 70 % at the literature cut off) underscores that it should not be used in isolation to determine patient management or prognosis after revision surgery for distal femur non-union. Rather, it serves as a valuable adjunct. It can help quantify risk based on readily available parameters, supplementing the surgeon's clinical assessment, interpretation of imaging and evaluation of biological factors associated with a higher risk of persisting non-union. Integrating the LEG NUI Score into the overall clinical picture has the ability to aid in counseling patients about their individualized risk profile and may help guide decisions regarding the intensity and frequency of postoperative follow-up. It might also contribute, alongside other factors, to discussions about the need for more aggressive biological augmentation or complex reconstructive strategies during the revision phase itself, although this study did not directly evaluate treatment modifications based on the score.

A striking finding in our study is the relatively low union rate of 55.6 % achieved after the index revision surgery for established distal femur non-union. This figure appears much lower than some reported overall success rates for distal femur non-union treatment in the literature, which can reach upwards of 80–90 % when considering the eventual union achieved after all necessary surgical procedures.^10^, ^11^, ^12^ Several factors likely contribute to this observed rate in our specific cohort and methodology. Firstly, our definition of success was strict, requiring radiographic union after only the first revision attempt performed at our institution for the established non-union. It did not capture patients who may have achieved union after a second or subsequent revision procedure. Furthermore, infected non-unions present a profound challenge to achieving union, typically requiring extensive debridement, targeted antibiotic therapy, and often staged procedures, all of which negatively impact healing rates after any single surgical intervention.^8^^,^^9^ Accordingly, some of the very high reported union rates are seen in cohorts exclusively made up of aseptic distal femur non-unions,^10^ whereas other cohorts including infected non-unions of the femur present with complication and repeat surgery rates as high as over 30 %.^8^^,^^12^

The LEG NUI score itself attempts to account for infection, but assigns it only a single point, which may not fully capture the magnitude of its detrimental effect on healing probability and likelihood for repeat surgery. Finally, our strict definition of union after the index revision inherently excludes the successful outcomes of planned multi-stage treatment protocols, such as the Masquelet technique or similar staged bone grafting procedures commonly employed for infected non-unions or those with large segmental defects.^11^^,^^12^ These techniques, by definition, require at least two distinct surgical procedures (implant/spacer placement and debridement, followed by later bone grafting) and would not be classified as achieving union after the “index revision surgery” according to our study's methodology. While these staged approaches can yield high rates of eventual union, their success is not reflected in our primary endpoint measurement. Therefore, the 55.6 % union rate should be interpreted specifically as the success rate of the initial surgical attempt to highlight the LEG NUI Score's applicability, rather than the overall final union rate achievable through potentially multiple procedures part of the non-union treatment process.

The study has several limitations. Overall patient number was low, reducing statistical power. Moreover, the inclusion of diverse non-union types (mechanical, biological, infected) introduces heterogeneity, making it challenging to isolate the score's performance independent of etiology without larger subgroup analyses. The cohort included patients with infected non-unions. Given that infection significantly impairs healing but contributes only one point to the LEG NUI Score, the score's ability to differentiate risk may be less accurate in populations with a high prevalence of infection, potentially underestimating the risk associated with this factor.^8^^,^^20^ Relying on retrospective data collection introduces potential for information bias (missing or inconsistently recorded data) and selection bias. Findings originate from a single institution, potentially limiting external validity and generalizability to settings with different patient demographics or surgical practices. The LEG NUI Score was initially validated for primary fracture fixation. Applying it to the more complex scenario of revision non-union surgery and in a distal femur loca represents an “off-label” use in terms of its original validation context. Its predictive accuracy in this specific setting needs external confirmation.

## 5 Conclusions

The LEG NUI Score demonstrates potential applicability beyond its original scope, showing utility in predicting healing outcomes following revision surgery for established distal femur non-unions. Calculation of the score may assist surgeons in risk-stratifying patients, helping to identify individuals who might warrant closer postoperative monitoring due to an elevated risk of persistent non-union. Such stratification could potentially optimize follow-up protocols and resource allocation. Nevertheless, definitive conclusions on its clinical value require further investigation. Larger, prospective validation studies are necessary to investigate these findings and determine the precise role of the LEG NUI Score in managing this challenging patient population.

## Declaration of generative AI and AI assisted technologies

Googles Gemini 2.5 Pro was used for AI assisted copy editing purposes. In this context, we define the term AI assisted copy editing as AI-assisted improvements to human-generated texts for readability and style, and to ensure that the texts are free of errors in grammar, spelling, punctuation and tone. These AI-assisted improvements may include wording and formatting changes to the texts. The authors claim full accountability for the final version of the text.

## Informed consent statement:

As only retrospective data was collected in accordance with the ethics committee decision, no separate consent for inclusion in the study database was required.

## Institutional review board statement:

The study was conducted in accordance with the Declaration of Helsinki and was approved by the local ethics committee (Nr. 850/2019BO2). The authors retrieved data from the TRUFFLE database (ClinicalTrials.gov NCT06098157).

## Author contributions

Conceptualization: BJB, CR, TM, SCH, MFRR, MMM, TH, MR; Methodology: BJB, CR, TM, MR; Data Curation: CR, MR; Investigation: BJB, CR, TM, MR; Formal Analysis: BJB, CR, TM, SCH, MFRR, MMM, TH, MR; Writing (First Draft): BJB, MR; Writing (Final Manuscript): BJB, CR, TM, SCH, MFRR, MMM, TH, MR. All authors have read and agreed to the published version of the manuscript.

## Ethics approval

The study was approved by the local ethics committee (Nr. 850/2019BO2). The authors retrieved data from the TRUFFLE database (ClinicalTrials.gov NCT06098157).

## Consent to participate/for publication

All patients consented to their surgical treatment. As only retrospective data was collected in accordance with the ethics committee decision no separate consent for inclusion in the study database was required.

## Funding

No funding concerning the study was received.

## Conflict of interest

BJB is consultant for BIOS Medical AG. There is no further conflict of interest.
